# The Rational Care and Treatment of the Insane

**Published:** 1912-02

**Authors:** A. W. Fly

**Affiliations:** Galveston, Member of State Board of Health


### The Rational Care and Treatment of the Insane.

BY A. W. FLY, M. D., GALVESTON, MEMBER OF STATE BOARD OF HEALTH.

The lunacy laws of the States of the United States are, for the most part, an incongruous mass of legal verbiage, incapable of explanation or judicial construction. They are in the main an elaboration of the laws of a century ago, with scarcely a recognition of the great advances which have been made in our knowledge of insanity. Notwithstanding the many improvements which have been made in providing for the physical comfort of the insane, there is, apparently, running through our lunacy laws the dominant idea of the criminality of insanity. Commitment and detention, and even care and treatment, are still subordinated to this ancient prejudice of our legislators. We are still governed by the common law of England, which declares that "it is only a person of unsound mind and dangerous to himself and others that may be restrained of his liberty by another." States are making better and better provisions for the security and well-being of the insane, while in custody, but they are doing absolutely nothing directly to restore the insane to health. Every improvement in asylum care is chiefly made for the purpose of safe detention, and not for cure. As a result, those in custody are constantly increasing, while the number of cures among them is at its minimum.

The whole drift of discussion, in regard to the insane, is in the direction of larger accommodations for the chronic insane. We scarcely ever hear of new methods of curing them, or great success in treatment. As a consequence, the States are burdened with taxation to provide for the accumulating masses of insane. Millions of dollars are annually expended to provide additional accommodations for the rapidly increasing chronic insane, and scarcely a farthing for the express purpose of effecting a speedy cure of the acute insane by remedial measures.

It is surprising how completely the one idea of the construction of asylums, and the methods of managing the details of their affairs, absorb even the best medical officers, and how little thought is given to the critical study of individual patients with a view to their prompt treatment and recovery. The reports of superintendents, of boards of trustees and lunacy commissioners abound with the details of new constructions, the results of good farming and gardening, the economies, the receipts and expenditures; but scarcely a word is said of the improved methods of treatment and the remarkable number of recoveries by new processes of treatment.

Two new facts are now almost universally recognized in our profession, if not universally acted upon by the public, as is certainly desirable:

1. That insanity is always a disorder or disease of the human organism.

2. That it is a disease that very speedily, if not taken at the onset, "takes a set" in the human system, or, as we say, becomes chronic and difficult, if not impossible to cure.

The general conviction of insanity as a disease is shown in the modern tendency to change the old name "lunatic asylums," to that of "Hospitals for the Insane" by legislative enactment. No longer should they be looked upon merely as places of custody and restraint of liberty, for fear of the harm their inmates may do when at large, but as institutions for treatment and cure of disease, with the application of medical science, and under the direction of the best medical skill and judgment to be had. With this hospital conception of our institutions, recognized by law, their privileges should be accessible to all the unfortunate victims of this disease as their right, on the shortest possible notice, without let or hindrance arising from the compliance with irksome, unnecessary and sometimes humiliating legal requirements. Immediate treatment is of higher obligation than any mere documentary and elaborate forms of law. The natural reluctance of the patient to admit his insanity, and the dread of publicity with a sense of shame, or disgrace, on the part of friends and family, are all sufficient causes of delay in a case, without adding to them a series of legal proceedings which, even to an ordinary outsider, bear too much the stamp of a criminal prosecution, and, in the ordinary patient, only serve to exacerbate his disease. Too much of our legislation seems to be based on the assumption of improper motives, on the part of friends and relatives, as if it were to be expected that cruelty and inhumanity, instead of being the rare exception, should be the normal rule and state of things, in civilized society. But a system of statutes founded on this supposition would be an insult, not only to the community in general, but especially to the profession. Self-interest, if no higher consideration, would make both family and physician alike chary of depriving a person of his liberty. No superintendent could have any motive to connive at an improper commitment, and even assuming the possibility of a corrupt official at the head of an institution, any conspiracy would have to involve many others, both inside and outside of the institution.

There are a vastly greater number of persons utterly lost to society and deprived of the capabilities of ordinary life by the shameful neglect and hindrance of those who should know better, in the first and early stage of this disease, than by all the mistakes of the undetected evil intentions of improper commitment than were ever known. If it had ever been possible to convince the public of this truth, so as by law to prevent that fatal procrastination with which many wait until more violent symptoms have set in, and if the vast mass of chronic insanity which now crowds our institutions had been put under that treatment within three months of the attack, it is safe to say that, at least, our present plans for the insane would hardly need enlargement. The saving of human lives and human minds to the community would have been enormous.

The question of detention is simply a medical question of the continuance of the care and treatment. In nearly every case we have ever known of a discharge on a writ of habeas corpus, the judicial certificate of insanity, whether arrived at by the court's personal observation or by witnesses of miscellaneous character before it, was such as might have replaced the medical certificate required by law, at the time of the patient's commitment.

In regard to the care of the insane, we may say the most notable advance of recent years, in our specialty, has been the establishment in many of our institutions of training schools for nurses and attendants, and the employment therein of those who have had the benefit of this special course of instruction. Wherever honestly tried, the results have practically exceeded the most sanguine anticipation.

Too much can not be said of the modern method of caring for the helpless insane on the congregate plan in large dormitories, day room and service rooms especially adapted for the management of this otherwise troublesome class of cases. In the first place, much has been gained by removing such patients from the general wards, where they were an eyesore and offense to their less demented fellows, and, in so far, a hindrance to recovery among the recoverable insane, who were forced into an association with them. Now, with suitable structural arrangements, and a special corps of nurses to wait upon such patients hand and foot, night and day, it is astonishing how much has been accomplished to create habits of order and cleanliness, and how much the general welfare of the whole hospital has been promoted.

The word "treatment" almost invariably suggests to the lay mind the idea of drugging. It is hard to dislodge the calomel and jap-lap of treating disease by the introduction of nauseous compounds into the corpus vile of the hapless patient. It is a relic of the same medieval mysticism that gave point to the caustic satire of Moliere's medical comedies, and enabled Sganarelle to expel his "peccant humors," and that, in recent times, has put much gold into the pockets of certain shrewd practitioners, however little of the precious metal may have found its way underneath the skin of the habitual drunkard. So great an authority as Dr. Clifford Allbut said (in discussing the proposed hospitals for the treatment of the insane, suggested by the London city council): "The fact is, gentlemen, this desire to reinstate treatment by medicine is a retrograde tendency. Modern therapeutics are marked, not by the multiplication of medicine, but by a gradual restriction of their field." And to those general practitioners whose cry is for more physic for the insane, he would put the awkward query, "Why they so constantly fail to cure their insane patients outside of an asylum, and how it happens that even those of them who have seen much of insanity are the first to impress upon people the futility of such outside treatment and the need of early removal to an institution for the insane?"

True, great advance has been made in the line of sedative and hypnotic remedies, as the result of painstaking research. Using the word "treatment" in its most comprehensive sense, the most marked progress has been made in securing for the patient the environment—physical, mental and moral—proper to his restoration. No longer the victim of drastic experiment in pharmacodynamics, greater attention is now paid to his diet and personal hygiene. He is better "groomed," if one may use the expression, than ever before. Conspicuous and pre-eminent among these modern methods of treatment is the provision of systematic and diversified occupation. The idea is probably as old as the disease itself. Sixty-five years ago Dr. Brigham, the first superintendent of the Utica State Hospital, a man far in advance of his age, had his shops in considerable variety, and his contemporary, that great man lately deceased, Pliny Earle, also promulgated sound views on this subject. So long ago as 1867 he ridiculed the idea of a mollycoddling optionalism in the application of this potent agency for the patient's cure and the deference of the physician, supposed to be rational, to the judgment of the patient known to be irrational.

Then, as now, there were patients who, at a certain stage of their disorder, could be cured by labor and apparently nothing else—patients who, in the absence of suitable occupation, become apathetic and incurable, and often dragged out their lives listless and imbecile by reason of the neglect to provide this kind of treatment. The query suggested itself to Dr. Earle, then: "Why it is that the only medicament which, as it is believed, will effect a cure is not prescribed and administered?" And this same question is today being propounded to progressive managers and superintendents all along the line. The answer may, perhaps, be found in the niggardliness of State legislatures to provide proper workshops for this kind of treatment, but more frequently I believe the explanation lies in the timidity of superintendents to urge, if not coerce, those patients whose objection lies in the often heard excuse: "I did not come here to work. If I can work here, I can work at home and be paid for my labor." Neither proposition is necessarily true, coming from the lips of an insane patient. As a matter of fact, much work can be done by patients under proper supervision in a well organized hospital that would be impossible of performance outside, and labor, like virtue, is its own reward.

I would here dwell upon the advisability of finding and obtaining sanction for the use of a market for the surplus produce of patients' labor and thus enable them to contribute to their own support while being maintained at public expense. Any hospital with a mass of able-bodied patients at its command can soon accumulate a surplus of mats, brooms, brushes, willow-ware and the like. The strong and fearless arm of philanthropy should protect institutions against a bullying at the hand of ignorant selfishness when organized labor interferes with the proper treatment of afflicted humanity.

Opportunity may also be taken here to refer more particularly to the great value of printing and bookbinding as avenues of useful and congenial labor for nimble-fingered women. This occupation meets very nicely the requirements in furnishing mental as well as physical stimulus to the insane. Hospitals for the insane might be permitted to do not only all their own printing, but also much of the printing required by the State elsewhere, and now done at public expense.

I am convinced that much saving could be accomplished to the State, and incalculable benefit provided for its patients, by establishing office and book bindery in every large institution for the insane throughout the country, provided always that such facilities as I have indicated be extended, either by the State or private encouragement. In thus extending the usefulness of shops, it would not be necessary to "breast and buffet breakers of public opinion." Public opinion would take up the cry, and such cruel demagogism as would antagonize the movement would meet with public execration. Then, the ghost once laid, there would be no excuse anywhere for failure to have recourse to restorative labor in every asylum in the land, and it would no longer be necessary for superintendents of hospitals to "anoint their consciences with the balsamic oil of belief that of two evils they choose the less."

When we reflect upon the effects soon discernible in the nervous system in men who retire from business late in life, often resulting in fatal collapse, it is entirely consonant that persons who have been accustomed to more or less steady occupation all their lives should not be completely cut off and sequestrated from it, so long as they have bodily vigor sufficient to be called into play. There can be no doubt that such exercise, both physical and mental, in due proportion, exerts a wholesome influence upon those parts of the organism which are disordered, and tends to the restoration of healthful functions.

In short, for the public mind to form a clear conception of what is best among all these points of treatment or hygienic management, it requires that the public mind should be more fully enlightened as to the nature of the disease of insanity itself, until it shall no longer be looked upon as a supernatural visitation, or some strange and unaccountable happening, for which confinement and restraint are the only remedy, but as a disease which has its causes either in the hereditary diathesis of the individual, or the accidents, abuses or the sanitary sins of life, and must be dealt with on the common sense principles and restitution and repair, when the evil has not passed the boundaries of a salvable vitality.

Again, the tendency is, nowadays, in view of the crowded condition of our institutions, to err, not on the side of caution, but rather to discharge patients from custody before their mental health has been fully re-established.

Here, of course, it will be pertinent to consider the functions of such boards as the American commissioners in lunacy. It is evident that the office of such boards should be in the main simply supervisory.

It is difficult to overestimate the benefit that may accrue to the service from exercise of a proper central supervision. But the too industrious exercise of detailed and minute executive powers would be bound to give rise to much friction and practical difficulties. The scope of medical judgment should be left as wide as possible, while the necessities of the mechanical plant and maintenance may, perhaps, derive vigor and completeness from outside direction.

If the superintendent is to be merely a medical clerk, subject to some remote central authority, the grade of such officers must suffer in the appointment of men whose ambition would scarcely be satisfied with such meagre opportunity for exercise of individual ability, skill and judgment, in the conduct of a hospital. It would have a tendency to cramp, rather than develop. A stunted uniformity is not the law of progress.

What Dr. Gooding has called "the day book and ledger plan" of caring for the insane, can never promote the growth of an enlightened system of public charities, though it may answer the ends of a centralized bureaucracy under an imperial government. This kind of thing almost always degenerates into a dull perfunctory round of duties, such as Dickens mercilessly caricatures under the symbol of red tape, in which nobody is supposed to know anything except what lies in his own immediate line.

It is particularly for this reason, also, that we must deprecate politics in asylum management as the greatest evil of the day, in the administration of State institutions. In the political world the emoluments of office have usually been regarded as of more importance than the responsibilities and work of the office itself. In the rivalry of political parties, it is extremely difficult to restore the moral standard of a true proportion between the honors and emoluments of office and the conscientious discharge of the work and duties expected of us.

In this matter of politics in public institutions, I regret to have to say that many States have furnished a palmary example of the mischief. The notion that medical officers of hospitals for the insane are the pensioners of a party, and not the servants of the whole people, seems to die hard.

We expose ourselves constantly to ridicule of the entire civilized world outside of our own country in seeming to entertain the idea that a man's political views has anything to do with his professional competency. It is the view that the salaries of these officers are not primarily the reasonable compensation for honest and faithful discharge of their professional duties, but the reward of their activity in entirely different fields that allows people to view, with indifference, such changes, entirely without regard to the merits, either of those who are turned out or those who are put in. The pernicious effect of such a policy is so plain that we should feel that we were insulting the intelligence of our hearers by arguing the question. Men whose aspirations are for professional eminence and usefulness will hesitate about accepting positions in which such qualities count for nothing. Even if competent men are secured, they are sure, in a State in which parties are pretty evenly balanced, under such a system, to be turned out before they have acquired the experience that will enable them to do their best work. The inevitable tendency, under such conditions, is to the filling of the offices by men whose only object is to make money out of them, and who, knowing that the time is short, will "make hay while the sun shines."

It is largely on the same lines that we may speak here of the board of managers or trustees of our State hospitals. The kind of men desirable for such positions are such as we would call men of affairs, with sufficient knowledge of what the public requires, and especially, the quality of benevolence and sympathy with common humanity. On no account should the officers be salaried. But it is not difficult to find men in every community willing to give their honorary services, as their contribution to the cause of charity. Moreover, it is a vast benefit to the various localities of the State, where these institutions are situated, to develop as widely as possible the local spirit of unselfishness and practical benevolence, and to enlist the interest and pride of many localities in the honor of the State, in support of its numerous institutions. The local managers, too, are able to gain a more intimate knowledge of the detailed affairs of such an institution, standing between the superintendent and the public, and are able to give such information to outside inquirers, in regard to internal conditions as an occasional visiting officer from a distance could not render. It is evident that the more sordid politician can have no use for such a place and position, or no claim upon the governor of the State, to appoint him to it. To constitute board of managers in such a manner would only be a stepping stone to fatal policy of abolishing such local trusteeship altogether, and establishing, in its place, a sort of official oligarchy at the seat of government to administer the charitable system of the State, on a plan too much assimilated to that of its penal system.

Also, there is some danger that, in the vast mass of chronic insanity, acute or recent cases may be neglected. The next step should be improved provision for the recoverable insane. The best efforts of the medical and nursing staff should be concentrated, in the hospital block, upon hopeful cases. The rude touchstone of expense should not, for a moment, be considered as a test of the desirability of such provision, but, on the contrary, nothing should be omitted, whether in construction or equipment, that makes for the recovery of our patients, in the incipiency of their disease. Such a service should embrace all the adaptable features of the modern general hospital, and have placed, at its disposal, the picked nurses of the institution. It should have facilities for classification sufficiently minute to provide, if necessary, for the isolation of individual cases. It might also contain an observation ward, into which all new cases, with few exceptions, could be first admitted. Preferably, such a hospital block should be situated near the main building, within easy access of the medical officers, so as to insure frequent visitation throughout the day. Given such enlightened provision, one might easily conceive of cases admitted to its wards and going on to rapid recovery, without subjecting them to a contact with the great mass of chronic insanity, which so often has a depressing effect upon recent cases.

In this connection, one might mention the out-patient department for mental cases in conjunction with the Pennsylvania Hospital for the Insane. This service was begun in October, 1885, in the building as the out-patient department of the Pennsylvania hospital, Philadelphia. It was suggested to Dr. Chapin by the fact that there were frequent applications for admission to the department for the insane of patients presenting an early physical impairment and nervous prostration culminating in insanity. They were mostly young men or women engaged in the mills and stores. It seemed that many of these persons, with proper attention and advice, could have escaped the attack of insanity. It was believed that, in a city with a population of 1,000,000, there were many cases of incipient insanity, if they could only be reached, that might be relieved without treatment in a hospital for the insane. In every dense population, there are, of course, many such cases. There is also a necessity for a place where information about the commitment of the insane, and about the insane, may be furnished. This service has been rendered by four assistant physicians of the Pennsylvania Hospital for the Insane. The number of new cases of the class, for whom the service is designed, that have come to this out-patient department, has averaged about thirty, yearly, and the number of visits made by each patient, six. Soon after the service was established, similar ones were begun in several other cities throughout the country. When the proposition to establish this out-patient department was first discussed, it was expected that a large number would apply, as it seemed it might meet a much-needed want. This expectation has not been entirely realized, yet it must be borne in mind, as an incident of the establishment, that a new department always grows slowly, regardless of its merit.

So the practical method of the question of the treatment of the insane, as in everything else, is the use of our science and common sense, in elevating ourselves and our fellowmen, on to higher planes of ethical life and living and removing all elements obstructive of effort in that direction, dealing with realities as they are, and not as imagination or pity would make them.—*Houston Post.*

